# Bronchoscopic lung volume reduction using an endobronchial valve to treat a huge emphysematous bullae: a case report

**DOI:** 10.1186/s12890-019-0849-z

**Published:** 2019-05-14

**Authors:** Eung gu Lee, Chin Kook Rhee

**Affiliations:** Division of Pulmonary, Allergy and Critical Care Medicine, Department of Internal Medicine, Seoul St. Mary’s Hospital, College of Medicine, The Catholic University of Korea, 222 Banpo-daero, Seocho-gu, Seoul, 06591 Republic of Korea

**Keywords:** Chronic obstructive pulmonary disease (COPD), Bullae, Bronchoscopic lung volume reduction (BLVR), Endobronchial valve (EBV)

## Abstract

**Background:**

In patients with chronic obstructive pulmonary disease (COPD), bronchoscopic lung volume reduction (BLVR) techniques using unidirectional endobronchial valves improve lung function and increase exercise tolerance. BLVR treatment is included in the Global Initiative for Chronic Obstructive Lung Disease (GOLD) treatment guidelines for COPD patients without interlobar collateral ventilation. However, BLVR using an endobronchial valve has not been attempted in patients with giant bullae.

**Case presentation:**

We report successful and safe BLVR using an endobronchial valve in a patient with a huge bullous emphysema in the right middle lobe. A 65-year-old male was diagnosed with COPD 5 years prior and had a large bullae in the right middle lobe at that time. During regular follow-up, the symptoms of respiratory distress gradually worsened, and the size of the bullae gradually increased on computed tomography (CT). Therefore, we decided to treat the patient via BLVR using an unidirectional endobronchial valve. The Chartis system (Pulmonx, Inc., Palo Alto, CA) confirmed the absence of collateral ventilation of the right middle lobe. We successfully inserted an endobronchial valve into the right middle bronchus. After insertion, the bullae decreased dramatically in size, and the patient’s symptoms and quality of life improved markedly.

**Conclusion:**

This case supports recent suggestions that BLVR can serve as a good alternative treatment for appropriately selected patients.

## Background

Chronic obstructive pulmonary disease (COPD) is characterized by persistent respiratory symptoms and airflow limitations caused by a combination of small airway disease (chronic bronchiolitis) and parenchymal destruction (emphysema) [1]; the latter involves permanent enlargement of the airspace in the distal parts of the terminal bronchioles because of destruction of the alveolar sacs. Emphysema triggers the loss of elastic tissue, airway collapse, and difficulties in gas exchange [2].

Standard treatments for COPD include smoking cessation, inhaled drugs such as long-acting beta-agonists and anti-muscarinic agents, pulmonary rehabilitation, and long term oxygen therapy. Lung volume reduction surgery (LVRS) improves the survival of patients with upper lobe-predominant emphysema and a low exercise capacity [1]. In such patients, LVRS affords a greater survival benefit than other medical treatments. However, non-upper lobe predominant emphysema is the sole predictor of operative mortality [3].

For selected patients with decreased lung function, advanced emphysema refractory to medical therapy or an inability to undergo LVRS, bronchoscopic lung volume reduction (BLVR) using an unidirectional endobronchial valve improves exercise tolerance and lung function [4, 5]. Valve implantation prevents hyperinflation by blocking inspiratory airflow to the targeted lung, and allows air to escape during exhalation. By reducing the size of a hyperexpanded emphysematous lung, the remaining lung re-expands and overall lung function is improved [6]. BLVR treatment is included in the Global Initiative for Chronic Obstructive Lung Disease (GOLD) treatment guidelines for COPD patients without interlobar collateral ventilation. Park et al. studied the relatively long-term outcomes of BLVR with endobronchial valve placement in Koreans with severe emphysema; the procedure proved safe and effective [7]. However, patients with giant bullae (greater than 5 cm in diameter) were excluded; no attempt to use BLVR/endobronchial valve placement to treat patients with giant bullae has yet been reported. Here, we report successful and safe BLVR using an endobronchial valve in a patient with a huge bullous emphysema in the right middle lobe.

## Case presentation

A 65-year-old male diagnosed with COPD 5 years prior was admitted to our hospital in November 2017. He had stopped smoking 2 years prior, but had a smoking history of 80 pack years. He had been taking indacaterol/glycopyrronium once daily and had been on 3.5 L/min home oxygen therapy for 2 years. In the past year, he had experienced two acute exacerbations that required hospitalization. A pulmonary function test (PFT) conducted in October 2017 revealed severe obstructive lung disease: the ratio of forced expiratory volume in 1 s (FEV1) to forced vital capacity (FVC) was 29%, the FEV1 was 0.41 L (percentage of predicted FEV1, 13%), the residual volume (RV) was 6.43 L (percentage of predicted RV, 275%); the total lung capacity (TLC) was 8.23 L (percentage of predicted TLC, 135%), and the percentage of predicted diffusing capacity of carbon monoxide (DLCO) was 23%. Arterial blood gas analysis revealed a pH of 7.413, PaCO2 of 53.8 mmHg and PaO2 of 65.4 mmHg.

**Fig. 1:**
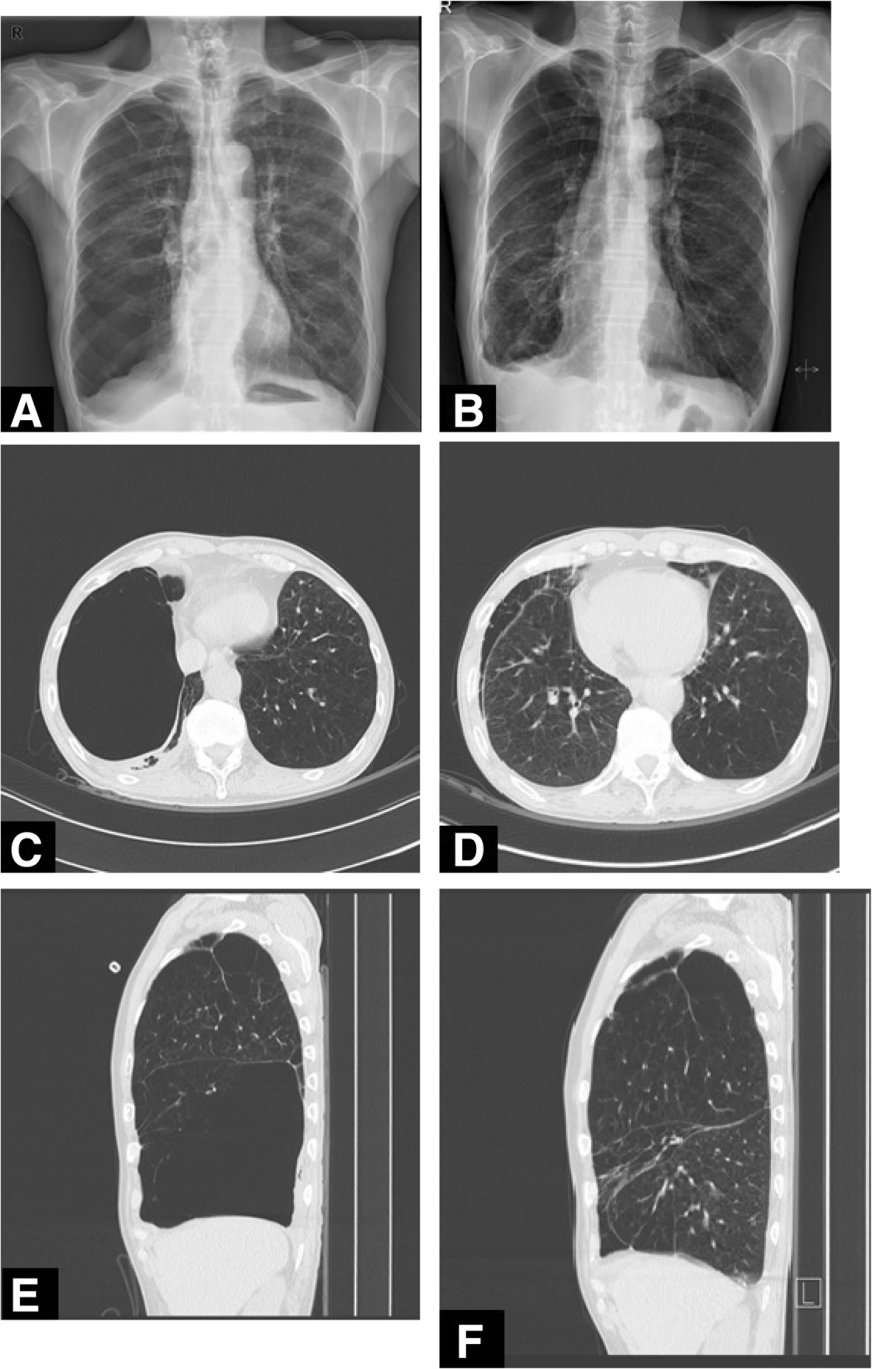
**a**, **c** and **e** Chest X-ray taken before the procedure (November, 2017) and computed tomography (CT) scan taken in May, 2017 (transverse and sagittal views, respectively) indicated severe emphysema and a huge bullae in the right middle lobe compressing the right lower lobe parenchyma. **b**, **d** and **f** A Chest X-ray taken 1 week after the procedure (November, 2017) and a CT scan taken 2 months after the procedure (January, 2018, transverse and sagittal views, respectively) showed that the huge bullae in the right middle lobe had disappeared. This caused the volume of the right middle lobe to decrease but that of the compressed right lower lobe to re-expand

Chest computed tomography (CT) performed in May 2017 indicated severe centrilobular emphysema in both lungs with huge bullae in the right middle lobe (Figs. 1c and e). The maximum area of the huge bullae in the axial view was 15.0 × 10.1 cm. On CT, the bullae became larger over time and the right lower lobe parenchyma became increasingly compressed. The fissure around the right middle lobe (the target lobe) was intact on chest CT.

We decided to perform BLVR using an unidirectional endobronchial valve. Atropine 0.5 mg was administered 30 min before bronchoscopy to minimize bronchial secretions. To locally anesthetize the oropharynx, we delivered 2 mL of lidocaine using a nebulizer. Commencing with bronchoscopy, we administered midazolam 3 mg for sedation and instilled lidocaine 10 mL for local anesthesia of the vocal cords and large airway. Using the Chartis system (Pulmonx, Inc., Palo Alto, CA), a catheter with a balloon at the distal tip was placed in the entrance of the right middle bronchus. The balloon was inflated to block the airway and the distal airflow, resistance, and pressure were measured. A gradual decrease in flow and increase in resistance and pressure were observed. Thus, we confirmed the absence of collateral ventilation of the right middle lobe [8]. The right middle bronchus was sufficiently small to be blocked by a single endobronchial valve; for this, we selected a Zephyr 5.5 endobronchial valve (Pulmonx).

After endobronchial valve insertion, chest X-rays were taken for 3 days and 1 week later, and confirmed that the size of the huge bullae had decreased dramatically (Fig. 1a and b). Two months after valve insertion, we performed chest CT, which showed that the endobronchial valve was located in the right middle bronchus, and that the huge bullae in the right middle lobe had disappeared. The volume of the right middle lobe had thus decreased but that of the compressed right lower lobe had re-expanded (Fig. 1d and f).

We performed a PFT at 2 months after valve insertion. The ratio of the FEV1 to FVC increased from 29 to 32%, and the FEV1 increased by 170 mL from 0.41 L (percentage of predicted FEV1, 13%) to 0.58 L (19%). The RV decreased from 6.43 L (percentage of predicted RV, 275%) to 4.74 L (201%), and the TLC decreased from 8.23 L (percentage of predicted TLC, 135%) to 6.61 L (108%). The symptoms and quality of life improved markedly, and no valve migration or obstruction, pneumonia, or pneumothorax has been noted to date.

## Discussion

Conventionally, the pathophysiology of bullous emphysema is considered to involve a valvular obstruction that allows air to enter, but not exit, the cystic space. However, a new hypothesis suggests that the bullous emphysema is associated with free airway communication caused by compliance higher than that of the surrounding lung [9]. For bullae that are not fed by an airway, endobronchial valve treatment will probably not be effective. However, in the present case, the size of bullae increased over time, indicating airway communication. Thus, when bullae are large, contained within a lobe, and likely fed by an airway, lobar volume reduction and reduction of the volume of bullae are essentially identical (i.e. the underlying mechanism/intention is the same).

Although differences in survival rates or long-term outcomes between patients undergoing BLVR and LVRS have not been documented, BLVR appears to effectively reduce mortality and improve the quality of life of patients who do not respond to medical treatment and are at high risk of surgical complications [5]. Klooster et al. found that endobronchial valve placement in appropriately selected patients significantly improved lung function and exercise capacity [5]. To optimize clinical outcomes, strict selection criteria are required when identifying patients who might benefit from BLVR [10]. Flandes et al. proposed the following criteria: symptomatic COPD; modified Medical Research Council (mMRC) dyspnea score > 1; emphysema-predominant phenotype; no more than two COPD exacerbations annually; severe airflow obstruction; hyperinflation and air trapping evident on the PFT; no smoking for at least 6 months, absence of prior lung surgery on the side of target lobe; and no collateral ventilation as confirmed by the Chartis system [11]. Our patient fulfilled all of these criteria so we performed lung volume reduction using an endobronchial valve after confirming the absence of collateral ventilation. Two case reports using BLVR to treat giant bullae in the right middle lobe have appeared worldwide. Tian et al. and Hou et al. successfully performed lung volume reductions using endobronchial valves in patients with giant bullae [12, 13]. Our results are superior, in that our patient had a lower basal pulmonary function and larger bullae, and exhibited greater improvement in pulmonary function, compared to the patients in the cited case reports.

## Conclusion

This case study supports recent suggestions that BLVR can serve as a good alternative treatment for appropriately selected patients.

## Abbreviations

BLVR: Bronchoscopic lung volume reduction
COPD: Chronic obstructive pulmonary disease
LVRS: Lung volume reduction surgery
PFT: Pulmonary function test
